# Novel Method for Rapid Assessment of Cognitive Impairment Using High-Performance Eye-Tracking Technology

**DOI:** 10.1038/s41598-019-49275-x

**Published:** 2019-09-10

**Authors:** Akane Oyama, Shuko Takeda, Yuki Ito, Tsuneo Nakajima, Yoichi Takami, Yasushi Takeya, Koichi Yamamoto, Ken Sugimoto, Hideo Shimizu, Munehisa Shimamura, Taiichi Katayama, Hiromi Rakugi, Ryuichi Morishita

**Affiliations:** 1 Department of Geriatric and General Medicine, Graduate School of Medicine, Osaka University, Suita, Osaka 565-0871 Japan; 2 Department of Clinical Gene Therapy, Graduate School of Medicine, Osaka University, Suita, Osaka 565-0871 Japan; 3 Department of Internal Medicine, Osaka Dental University, Hirakata, Osaka 573-1121 Japan; 4 Department of Neurology, Department of Health Development and Medicine, Osaka University, Suita, Osaka 565-0871 Japan; 5 Department of Child Development, United Graduate School of Child Development, Osaka University, Kanazawa University, Hamamatsu University School of Medicine, Chiba University, and University of Fukui, Suita, Osaka 565-0871 Japan

**Keywords:** Cognitive ageing, Alzheimer's disease

## Abstract

A rapid increase in the number of patients with dementia has emerged as a global health challenge. Accumulating evidence suggests that early diagnosis and timely intervention can delay cognitive decline. The diagnosis of dementia is commonly performed using neuropsychological tests, such as the Mini-Mental State Examination (MMSE), administered by trained examiners. While these traditional neuropsychological tests are valid and reliable, they are neither simple nor sufficiently short as routine screening tools for dementia. Here, we developed a brief cognitive assessment utilizing an eye-tracking technology. The subject views a series of short (178 s) task movies and pictures displayed on a monitor while their gaze points are recorded by the eye-tracking device, and the cognitive scores are determined from the gaze plots data. The cognitive scores were measured by both an eye tracking-based assessment and neuropsychological tests in 80 participants, including 27 cognitively healthy controls (HC), 26 patients with mild cognitive impairment (MCI), and 27 patients with dementia. The eye tracking-based cognitive scores correlated well with the scores from the neuropsychological tests, and they showed a good diagnostic performance in detecting patients with MCI and dementia. Rapid cognitive assessment using eye-tracking technology can enable quantitative scoring and the sensitive detection of cognitive impairment.

## Introduction

With the increase in the number of patients with dementia, the growing economic impact related to the diagnosis and treatment of dementia has become a global issue^1^. Although a cure for dementia is yet to be established^2,3^, recent studies have shown that early intervention through an improvement in lifestyle behaviors or actively engaging in exercise therapy can delay the progression of cognitive impairment^1,4,5^. Early diagnosis and timely intervention during the pre-dementia phase, known as mild cognitive impairment (MCI), or in the early stages of dementia are key to tackling dementia.

The first step toward diagnosing dementia begins with a cognitive function assessment using a neuropsychological test, which generally consists of asking questions via traditional “pen-and-paper” tests. The Mini-Mental State Examination (MMSE) is a commonly used assessment tool for detecting cognitive decline, which can be administered by physicians or general practitioners^6,7^. MMSE scores, which reflect the subject’s global cognitive function, are clinically useful, with well-established utility^8,9^. Therefore, the MMSE, together with other neuropsychological tests, such as the Alzheimer’s Disease Assessment Scale-cognitive subscale (ADAS-Cog) and the Frontal Assessment Battery (FAB), has been used in most of the clinical research and clinical trials as a primary cognitive endpoint^10^.

While these neuropsychological tests are valid and reliable, they have some limitations as screening tools for dementia. The MMSE requires approximately 10–20 min to complete and tends to take longer when administered to elderly patients^11^. The ADAS-Cog, a more accurate assessment of the severity of the cognitive impairment, requires approximately 30–45 min^12^. Subjects may experience high levels of psychological stress, as they are supposed to answer a series of questions during the assessment. These traditional neuropsychological tests are not simple, and neither are they sufficiently brief for routine MCI and dementia screening in a clinic or at a population level. Rapid and practical assessments are needed to tackle the global epidemic of dementia^1^.

Another potential issue regarding the traditional neuropsychological tests is reproducibility. Highly trained clinical neuropsychologists are needed to administer these tests properly, since the rater’s level of proficiency can affect the results. Motor impairment, such as Parkinsonism or post-stroke paralysis, which is often accompanied in patients with dementia, can affect the results of these cognitive tests, as writing and drawing are required in a portion of the tests.

Eye tracking is a new technology to measure eye movement and the subject’s gaze positions objectively^13^. Generally, an infrared camera light source is used to detect the position of the subject’s pupils and determine the gaze points using some mathematical algorithms. The eye-tracking technology enables quantitative and objective assessment of eye movements in a non-invasive manner, which can be applied to neuroscience research to assess cognitive function or impairment, such as traumatic brain injury, autism spectrum disorder, and neurodegenerative diseases^13–27^.

Here, we developed a novel brief and practical cognitive assessment tool using a high-performance eye-tracking technology with short task movies and pictures to assess cognitive function. The subject simply views a series of short movies and pictures displayed on a monitor for 178 s while their gaze points are recorded by the eye tracking device. Each task is designed to assess specific neurological domains, including deductive reasoning, working memory, attention, and memory recall. In each task, multiple images, including a correct answer (target image) and distractors (incorrect non-target images), are presented on the display, and the subject is instructed to identify and focus on the correct answer. A region of interest (ROI) is set on the correct answer, and the cognitive scores are determined from the gaze plot data by measuring the fixation duration on the ROI of the target image (see Methods and Supplemental Information for details). The percentages of fixation duration from each task are averaged and used as an eye tracking–based cognitive score.

We measured eye tracking-based cognitive scores in cognitively healthy controls (HC), patients with MCI, and patients with dementia and evaluated the correlation with the cognitive scores from the conventional neuropsychological tests. The eye tracking-based cognitive scores correlated well with the scores from the MMSE and other neuropsychological tests and showed a good diagnostic performance in detecting cognitive impairment in patients with MCI and dementia.

## Results

### Participant characteristics

Demographic characteristics of the participants are shown in Table 1. The cognitive function of HC (n = 27), patients with MCI (n = 26), and patients with dementia (n = 27) were assessed. HC tended to be younger than patients with MCI and those with dementia, though the difference did not reach statistical significance. The gender distribution did not differ among the groups. The mean MMSE scores were 28.7 in HC, 25.7 in patients with MCI, and 16.0 in patients with dementia, showing a statistically significant difference (Kruskal-Wallis test (H = 58.3, df = 2, Cramer’s V = 0.19, *p* < 0.0001) followed by Steel-Dwass multiple comparison test (*p* < 0.01)). A subset of participants underwent other cognitive assessments, including the ADAS-Cog, FAB, and Clinical Dementia Rating (CDR); patients with MCI and dementia performed significantly worse than HC.

**Table 1:** Participant characteristics.

	HC	MCI	Dementia	*p* value
No.	27	26	27	
Age, years, mean (SD)	71.5 (11.1)	75.2 (8.2)	75.4 (9.5)	0.40
Sex, male, n (%)	9 (33.3)	9 (34.6)	11 (40.7)	0.83
MMSE, mean (SD)	28.7 (1.6)	25.7 (3.0)_a_	16.0 (4.4)_a,b_	<0.0001
FAB available	5	18	18	
FAB, mean (SD)	13.6 (1.8)	13.4 (2.4)	9.9 (2.7)_b,c_	<0.001
ADAS-Cog available	3	14	17	
ADAS-Cog, mean (SD)	4.4 (1.3)	9.4 (3.4)	18.7 (5.9)_b,c_	<0.0001
CDR available	11	13	19	
CDR, mean (SD)	0 (0.0)	0.5 (0.2)_a_	1.0 (0.6)_a,b_	<0.0001

Age and MMSE, FAB, ADAS-Cog, and CDR scores were compared by the Kruskal-Wallis test followed by Steel-Dwass multiple comparison tests. The sex ratio was analyzed using the Chi-square independence test. a p < 0.01 compared to HC, b p < 0.01 compared to MCI, c p < 0.05 compared to HC. HC, Healthy controls; MCI, mild cognitive impairment; ADAS-Cog, Alzheimer’s Disease Assessment Scale-cognitive subscale; FAB, Frontal Assessment Battery; MMSE, Mini–Mental State Examination; CDR, Clinical Dementia Rating.

### Rapid cognitive assessment using an eye-tracking system and task movies and pictures

**Figure 1:**
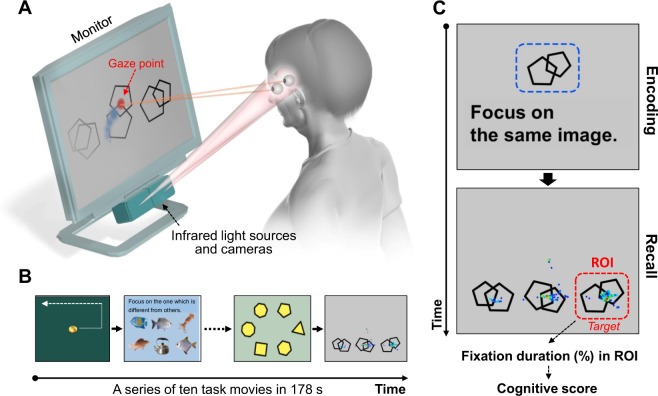
Rapid cognitive assessment using an eye-tracking system and tasks. (**A**) The eye tracking system used in the study. The gaze point of the subject was recorded using infrared light sources and cameras located below the monitor. (**B**) A series of ten task movies and pictures (178 s in total) is displayed on the monitor, and the subject views them. Four representative task movies and pictures are shown. Each task assesses eye movement, deductive reasoning (odd-one-out task), visuospatial function, and working memory (from left to right). Images (gold coin, fishes, and kettle) are obtained from JVC KENWOOD Corporation with permission. (**C**) An example of a working memory task and representative gaze plots with a duration-based heatmap obtained from a control subject. Gaze plots represent the location and time spent looking at the objects. A cue object (double pentagon) is presented for 10 s (encoding), followed by three distinct objects with the same one as the cue object (right bottom on the monitor, *target*). The subject is asked to remember and gaze at the target object. Fixation duration within the region of interest (ROI) set on the target object was used as a measure of the cognitive score. Full details of the procedure are described in the Supplemental Information.

Figure 1A shows the eye tracking-based cognitive assessment system used in the study. A series of ten task movies and pictures (178 s in total) is displayed on the monitor (Fig. 1B), and the subject views them. The subjects were instructed to remember and focus on the target subject (correct image). The percentage of time the subject spent focusing on the target image (% fixation duration within the ROI) was used as a measure of the cognitive score (Fig. 1C). Details of the procedure are described in the Methods and Supplemental Information.

### Positive correlation between MMSE scores and the eye tracking-based cognitive scores

**Figure 2:**
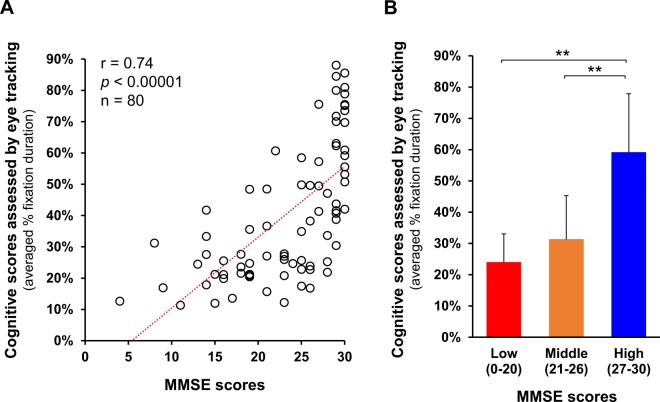
Correlation between MMSE scores and cognitive function scores, as assessed by the eye-tracking system. (**A**) Cognitive scores assessed by eye tracking system showed a strong positive correlation with the MMSE scores. *p* < 0.00001, Spearman’s rank test, n = 80. (**B**) Subjects were divided into three MMSE categories based on the severity; Low, 0–20/30 (severely impaired, n = 24); Middle, 21–26/30 (mildly impaired, n = 22); High, 27–30/30 (no apparent impairment, n = 34). Subjects in Low and Middle MMSE score categories showed lower cognitive scores assessed by eye tracking system. **p < 0.01, ANOVA followed by the Tukey–Kramer multiple comparisons test. Error bars represent standard errors.

All 80 participants underwent both the MMSE and an eye tracking-based cognitive assessment. Figure 2A shows the scattered plots of the MMSE scores and the scores assessed by the eye-tracking system (average % fixation duration on the target image from all task movies). The cognitive score assessed by the eye tracking system showed a strong positive correlation with the MMSE score (Fig. 2A, r = 0.74, *p* < 0.00001, Spearman’s rank test).

Subjects were divided into three MMSE categories based on severity: Low, 0–20/30 (severely impaired, n = 24); Middle, 21–26/30 (mildly impaired, n = 22); and High, 27–30/30 (no apparent impairment, n = 34). Subjects in the Low and Middle MMSE score categories performed significantly worse than those in the High MMSE category in the eye tracking-based cognitive assessment (Fig. 2B, ANOVA ((F(2, 77) = 43.9, η^2^ = 0.53, *p* < 0.01) followed by the Tukey–Kramer multiple comparison test (*p* < 0.01)).

### Diagnostic performance of the eye tracking-based cognitive assessment

**Figure 3:**
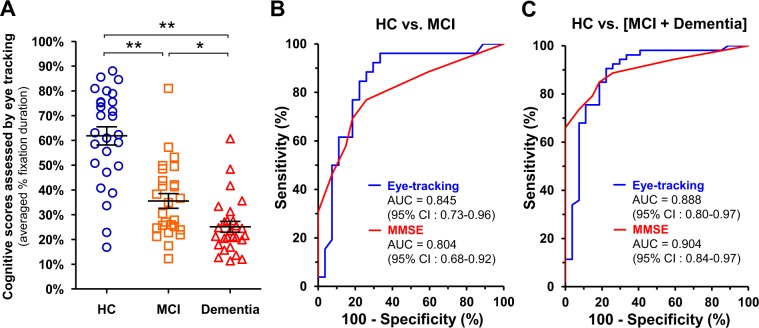
Diagnostic performance of the eye tracking-based cognitive assessment. (**A**) Cognitive scores assessed by the eye tracking system in clinical subgroups: HC (n = 27), MCI (n = 26), and dementia (n = 27). *p < 0.05, **p < 0.01, ANOVA followed by the Tukey–Kramer multiple comparisons test. Error bars represent SEM. (**B**,**C**) ROC curve analysis of diagnostic performance of the eye tracking-based cognitive assessment (blue) and the MMSE (red) for discriminating MCI from HC (**B**) and any type of cognitive impairment [MCI + dementia] from HC (**C**). The area under the ROC curve (AUC) analysis was used to compare the diagnostic performance of the eye-tracking cognitive test and the MMSE. HC, Healthy controls; MCI, mild cognitive impairment; ROC, receiver operating characteristic.

We next examined the diagnostic performance of the eye tracking-based cognitive assessment (Fig. 3). Subjects were classified based on their clinical categorization as HC, MCI, and dementia. Patients with MCI and dementia had statistically significantly lower scores than HC in the eye tracking-based cognitive assessment (Fig. 3A, ANOVA ((F(2, 77) = 40.0, η^2^ = 0.51, *p* < 0.01) followed by the Tukey–Kramer multiple comparison test (*p* < 0.01)), and patients with dementia performed worse than MCI (Fig. 3A, ANOVA ((F(2, 77) = 40.0, η^2^ = 0.51, *p* < 0.01) followed by the Tukey–Kramer multiple comparison test (*p* < 0.05)). The receiver operating characteristic (ROC) curve analysis was used to assess the accuracy of the eye tracking-based cognitive assessment for diagnosing MCI or dementia (Fig. 3B,C). To discriminate patients with MCI from HC, the eye tracking-based assessment archived an are under the curve (AUC) of 0.845 (95% CI 0.73–0.96), which was comparable with the MMSE (AUC = 0.804, 95% CI 0.68–0.92) (Fig. 3B). The eye tracking-based assessment showed a good accuracy in discriminating patients with any type of cognitive impairment (MCI + dementia) from HC (AUC = 0.888, 95% CI 0.80–0.97), which was also comparable with the MMSE (AUC = 0.904, 95% CI 0.84–0.97) (Fig. 3C).

### Correlation with other neuropsychological assessments

**Figure 4:**
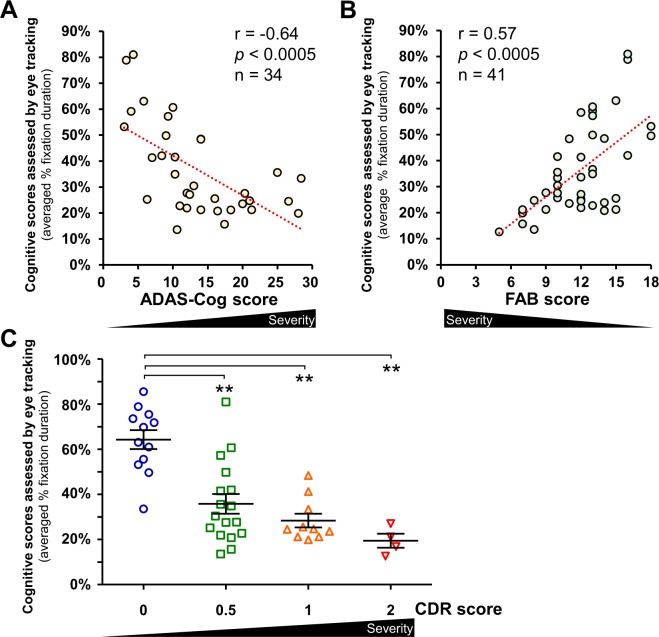
The cognitive scores assessed by the eye tracking system correlates with ADAS-Cog, FAB, and CDR scores. (**A**) The correlation between ADAS-Cog scores and the cognitive scores assessed by the eye tracking system. *p* < 0.0005, Spearman’s rank test, n = 34. (**B**) The correlation between FAB scores and the cognitive scores assessed by the eye-tracking system. *p* < 0.0005, S*p*earman’s rank test, n = 41. (**C**) The correlation between CDR scores and the cognitive scores assessed by the eye tracking system. **p < 0.01, ANOVA followed by the Tukey–Kramer multiple comparisons test, n = 43. Error bars represent SEM. ADAS-Cog, Alzheimer’s Disease Assessment Scale-cognitive subscale; FAB, Frontal Assessment Battery; CDR, Clinical Dementia Rating.

The cognitive scores assessed by the eye tracking system correlate well with the scores from the ADAS-Cog (Fig. 4A) and FAB (Fig. 4B). The subjects with cognitive impairment with a higher ADAS-Cog score had lower scores in the eye tracking-based assessment (Fig. 4A, r = −0.64, *p* < 0.0005, Spearman’s rank test, n = 34). Meanwhile, the subjects with impaired frontal lobe function with a lower FAB score had lower scores in the eye tracking-based assessment (Fig. 4B, r = 0.57, *p* < 0.0005, Spearman’s rank test, n = 41).

The eye tracking-based score correlated with the CDR scores, which reflects the degree of dementia severity (Fig. 4C, ANOVA ((F(3, 39) = 16.0, η^2^ = 0.55, *p* < 0.01) followed by the Tukey–Kramer multiple comparison test (*p* < 0.01)). Notably, subjects with a CDR score of 0.5, who are at a high risk of developing dementia, had significantly lower scores in the eye tracking-based assessment (Fig. 4C, *p* < 0.01), suggesting a high sensitivity for detecting patients in a very early stage of dementia.

## Discussion

Given the recent evidence demonstrating the benefits of early diagnosis and timely intervention for dementia^1,5,28,29^, there is a heightened need for a rapid and practical cognitive assessment tool that can be used in routine clinical practice and at a population level. The traditional neuropsychological tests, such as MMSE and ADAS-Cog, are well validated for the detection of dementia; however, they take time (longer than 10–20 min.) to complete and they require a highly trained examiner to obtain reliable scores. Furthermore, the psychological burden during the assessment and score variability, due to examiners’ varying levels of proficiency, can also result in a bottleneck situation in the application of these neuropsychological tests for routine screening. In this study, we demonstrated the utility of eye tracking-based cognitive assessment as a screening tool for the early detection of cognitive impairment. Using a high-performance eye tracking technology in combination with short task movies and pictures, cognitive scores, which were highly correlated with traditional neuropsychological tests, were obtained in a short period of time (178 s).

The task used in this study were a series of 10 short movies and pictures; each was designed to assess specific neurological domains, including memory encoding, deductive reasoning, visual working memory, attention and calculation, and memory recall (Fig. A and Methods “Task movies and pictures for the assessment of cognitive function”). The subject’s average score for all tasks was used in the analysis, displaying significant correlations with the scores of multiple cognitive tests, including MMSE, ADAS-Cog, FAB, and CDR (Figs 2, 4), suggesting that the eye tracking-based cognitive score reflects the subject’s global cognitive function. The degree of correlation with the MMSE scores varied among the individual task, with the highest correlation occurring with respect to the memory recall task (Task 1-b), followed by the two attention and calculation tasks (Task 6 and 7), and the deductive reasoning task (Task 3) (Supplemental Fig. 1). This implies that tasks can be customized for the specific aims and requirements of a specific study. For example, tasks customized for memory recall may be useful in detecting Alzheimer’s disease, which is characterized by progressive memory loss.

Notably, the eye tracking-based assessment achieved a high diagnostic performance for discriminating MCI, which was comparable with the MMSE (Fig. 3B), suggesting the utility of this approach in the detection of very early-stage dementia. Recent studies have reported that a significant portion (up to 30%) of all dementia cases can be attributed to modifiable risk factors, such as diabetes and hypertension; multidomain intervention is expected to reduce one’s risk of developing dementia^1,30^. Early diagnosis can give individuals with cognitive decline the opportunity to benefit from symptomatic treatments such as cholinesterase inhibitors^31,32^. Additionally, the early detection of cognitive impairment may increase the efficiency of patient recruitment for clinical trials of Alzheimer’s drug development, which has shifted to focus on the early stage of the disease^33^.

Due to the simplicity of this procedure, this approach has high usability and acceptability rates, even for very elderly patients with severe cognitive impairment. Elderly patients with dementia may have increased difficulty or experience the psychological burden of concentrating on a particular cognitive test for a long period of time. In contrast, the eye tracking-based assessment requires minimal effort from patients, simply requiring that they be seated in front of a monitor and view the short movies and pictures; this may be the principle advantage of this method in its use as a screening tool for elderly patients with cognitive impairment. Indeed, neither patients’ age nor the severity of their cognitive impairment, as measured by the MMSE, affected the recording efficiency of this eye-tracking assessment (Supplemental Fig. 2A,B). On the other hand, eye tracking-based cognitive assessment depends on the intact visual function of the subject, which is one limitation of this approach. For example, although eyeglasses had little impact on recording efficiency, eye tracking proved to be unsuccessful in patients with strabismus. Although we did not include such participants in the cohort of this study, severe cataracts, another common comorbidity in elderly patients with cognitive impairment, may affect the efficiency of eye tracking assessments. The possible effects of hearing loss appeared to be minimal, since written instructions are presented on the monitor during the assessment (see Methods “Task movies and pictures for the assessment of cognitive function” and Supplemental Information).

Previous studies using eye-tracking technology have demonstrated oculomotor alterations in patients with AD, including abnormalities in saccade latencies^18–20^ and accuracy^21–23^. Additionally, some researchers have reported the potential usefulness of eye-tracking metrics for assessing higher order cognitive functions. Pavisic *et al*. reported that individuals with young onset AD had abnormal eye movement patterns in fixation, stability, saccade, and smooth pursuit tasks as compared to controls^24^, providing evidence that eye-tracking metrics could be useful for detecting higher order cognitive impairments. Other researchers have also demonstrated that eye movement metrics could potentially predict memory deficits in patients with dementia^25–27^. These findings suggest that eye tracking–based assessment could be helpful in the differential diagnosis of dementia. Another important aspect of the diagnosis of dementia is an evaluation of the severity of cognitive deficits, since this helps with accurate diagnosis, assessment for response to symptomatic treatments, and prognosis. In this study, we successfully demonstrated a strong correlation between scores from eye-tracking measurements and well-validated neuropsychological tests (Figs 2A, 4). The simplicity of the eye-tracking system used in this study could be another potential advantage over the above-mentioned studies. We used an all-in-one recording device with no requirements for rigid head-stabilization or head-mounted devices, which is suitable for a screening test.

The eye tracking-based approach requires minimal instruction by examiner; only a brief explanation regarding the procedure prior to the assessment with no spoken instruction given during the tasks. This provides advantages including; i) minimal requirement for rater training and qualification, ii) ensuring consistent administration of the assessment, and iii) increasing the reproducibility of the results. Objective and automated scoring algorithm that is based on the gaze plots data creates additional advantages, enabling the efficient analysis of large datasets, the unbiased interpretation of results, and the production of a standardized assessment tool with minimal cofounding variables. The limitations of the eye tracking–based cognitive assessment include cost and practicality concerns. Although there are minimal requirements for staff training, this system requires a high-performance eye-tracking device, which could be a potential barrier for the wide availability of this system.

In summary, we developed a novel rapid screening tool for cognitive impairment using a high-performance eye tracking technology with short task movies and pictures. This method enables the quick and highly sensitive semi-automated assessment of patients’ cognitive function with minimal potential examiner issues, overcoming some of the limitations of traditional neuropsychological tests, as a routine dementia screening. This simple and repeatable assessment tool may be useful as a supplement for well-validated neuropsychological tests and in longitudinal studies intended to measure and monitor the influence of intervention treatments.

## Methods

### Subjects

The study cohort included 27 HC, 26 patients with MCI, and 27 patients with dementia recruited at Osaka University Hospital. Physicians specialized in geriatric medicine and dementia assessed all participants. All subjects underwent standard physical and neurological examinations, neuropsychological assessments, and brain MRI scans within six months of the eye-tracking evaluation. Subjects with MCI and dementia underwent standard blood tests to rule out other disorders that may cause cognitive impairment, such as thyroid disorder or vitamin deficiencies. The patients with MCI met the revised Petersen criteria^34^, and the patients with dementia met the dementia criteria defined by the DSM-IV^35^. For the HC group, we recruited individuals who had no active neurologic or psychiatric diseases, with normal cognitive function, an MMSE score between 25 and 30, and a CDR score of 0. Participant demographics are provided in Table 1.

The MMSE and ADAS-Cog were used to assess global cognitive function. The MMSE scores range from 0 to 30, with lower scores indicating greater impairment. The ADAS-Cog scores range from 0 to 70, with higher scores indicating greater impairment. The FAB was used to examine executive functions. The FAB scores range from 0 to 18, with lower scores indicating greater impairment. The CDR scale was used to assess the severity of cognitive impairment. A CDR of 0 indicates no cognitive impairment; a CDR of 0.5 indicates MCI or the very early stage of dementia; and CDRs 1, 2, and 3 indicate dementia with increasing severity. The eye tracking-based assessment and neuropsychological tests were performed by independent physicians who were blinded to the results of each other’s assessments.

All participants and their families provided written informed consent. All study protocols were approved by the Osaka University Hospital Institutional Review Board, and ethical approval was provided by the Ethics Committee of Osaka University. All research was performed in accordance with relevant guidelines and regulations including the Declaration of Helsinki.

### Measurement of gaze points

Participants’ gaze plots were recorded using a high-performance eye-tracking device (Gazefinder NP-100, JVC KENWOOD Corporation, Kanagawa, Japan), as described previously^13,15,17^. Briefly, the device uses infrared light sources and cameras, which are located under a 19-inch monitor (1280 × 1024 pixels), to detect the subject’s eye position using corneal reflection techniques. The gaze points were recorded at a frequency of 50 Hz while the task movies and pictures (see below) were displayed on the monitor.

### Task movies and pictures for the assessment of cognitive function

The subject is seated in front of a monitor and instructed by the examiner to view the movies and pictures displayed on it. Both spoken (by the computer) and written (on the monitor) instructions are presented prior to or during some of the task movies and pictures, while the examiner gives no instruction during the tasks. After a brief calibration of eye position (approximately 20 s)^13^, a series of ten task movies and pictures are presented on the monitor as follows (see also Supplemental Information):

#### Task 1-a: Memory task (encoding) (14 s)

A bear and five different types of foods on a table are displayed on the monitor. A bear eats one of the foods and the subject is asked to remember which one the bear ate. Both spoken and written instructions are presented during the task movie.

#### Task 2: Assessment of smooth pursuit eye tracking (11 s)

A coin moves around on the monitor and the subject tracks the moving coin using his/her eyes. This movie is for the qualitative assessment of eye movement itself and for the exclusion of subjects with gaze palsy, and the gaze plot data was not used for scoring cognitive function in the current study. Neither spoken nor written instructions are presented in this task.

#### Task 3: Deductive reasoning (odd one task) (10 s)

Six different objects are displayed on the monitor, and the subject focuses on the one that differs from the others. Both spoken (by the computer) and written (on the monitor) instructions are presented just before the task.

#### Task 4: Visual working memory task 1 (pattern matching) (20 s)

In the first part, an object, which is a combination of a circle and a triangle, is displayed on the monitor, and the subject is instructed to remember it by both oral and written instructions. In the following part, four different objects are presented on the monitor and the subject focuses on the same one that was presented in the first part.

#### Tasks 5–7: Attention and calculation task (56 s in total)

In the first part, different numbers of apples and bananas are presented on the monitor and the subject is instructed to count them. In the following part, the subject is asked to subtract the number of bananas from the number of apples and to find the answer among eight distinct numbers presented on the monitor. The number of objects (total number of apples and bananas) presented on the monitor increases from task 5 to task 7 with rising difficulty (from easy to difficult).

#### Tasks 8–9: Visuospatial function task (24 s in total)

In the first part, the subject is instructed to focus on the pentagon (task 8) or hexagon (task 9) by both oral and written instructions. In the following part, five distinct objects, including a pentagon, are presented on the monitor.

#### Task 10: Visual working memory task 2 (intersecting double pentagon) (20 s in total)

In the first part, an intersecting double pentagon is displayed on the monitor, and the subject is instructed to remember it by both oral and written instructions. In the following part, three double pentagons with distinct intersecting orientations are presented on the monitor, and the subject must focus on the same one that was presented in the first part.

#### Task 1-b: Memory task (recall) (10 s)

The bear and the five different types of foods that were presented in task 1-a are again displayed on the monitor (154 s after encoding task 1-a). The subject is asked to focus on the food that the bear ate in task 1-a.

### Data analysis for cognitive score

An ROI was set on the correct answer (target image), and the % fixation duration on the ROI was used as a measure of cognitive score (see Fig. 1C). The time for which valid gaze plots were successfully detected, not the total task duration, was used as the denominator for % fixation duration on the ROI, taking account of data tracking loss due to blinking or looking away from the monitor. Data regarding the percentage of successful gaze detection for each subject during the whole task is shown in Supplemental Fig. 2. We simply measured the periods when gaze plots fell on the ROI, but saccades and fixations were not strictly distinguished in this analysis. Representative heatmap images obtained from HC and dementia are shown in Supplemental Fig. 3. The results of % fixation time duration from nine tasks (tasks 3, 4, 5, 6, 7, 8, 9, 10, and 1-b) were averaged (without being weighted across the tasks) and used as an eye tracking–based cognitive score.

### Statistical analysis

Difference in participants’ age, MMSE, FAB, and ADAS-Cog scores among the three groups were analyzed by the Kruskal–Wallis test followed by the post-hoc Steel–Dwass test for multiple comparison. The two-sided Chi-square test was used to test frequency differences among the groups. A comparison of eye tracking-based cognitive scores among the groups was performed by one-way ANOVA followed by the Tukey–Kramer test. Spearmen’s rank correlation was applied to determine the correlation between the eye tracking-based cognitive scores and the results of the neuropsychological tests (MMSE, ADAS-Cog, and FAB). The diagnostic performances of the eye tracking-based cognitive assessment and the MMSE were determined using a ROC analysis. An area under the ROC curve was used as an index of diagnostic performance for discriminating MCI or any type of cognitive impairment [MCI + dementia] from HC.

Statistical analysis was performed using the Statcel 4 (OMS Publishing Inc., Tokorozawa, Japan) and GraphPad Prism 5 (GraphPad Software Inc., San Diego, CA) software. The results are expressed as the mean ± SD or SEM, as indicated in each figure legend. *P* < 0.05 was considered significant.

## Supplementary information

Supplemental information

## References

[1] Livingston G (2017). Dementia prevention, intervention, and care. Lancet.

[2] Yan R, Vassar R (2014). Targeting the beta secretase BACE1 for Alzheimer’s disease therapy. Lancet Neurol.

[3] Karran E, De Strooper B (2016). The amyloid cascade hypothesis: are we poised for success or failure?. J Neurochem.

[4] Karssemeijer EGA (2017). Positive effects of combined cognitive and physical exercise training on cognitive function in older adults with mild cognitive impairment or dementia: A meta-analysis. Ageing Res Rev.

[5] Ngandu T (2015). A 2 year multidomain intervention of diet, exercise, cognitive training, and vascular risk monitoring versus control to prevent cognitive decline in at-risk elderly people (FINGER): a randomised controlled trial. Lancet.

[6] Folstein MF, Folstein SE, McHugh PR (1975). “Mini-mental state”. A practical method for grading the cognitive state of patients for the clinician. J Psychiatr Res.

[7] Lin JS, O’Connor E, Rossom RC, Perdue LA, Eckstrom E (2013). Screening for cognitive impairment in older adults: A systematic review for the U.S. Preventive Services Task Force. Ann Intern Med.

[8] Hopp GA, Dixon RA, Grut M, Backman L (1997). Longitudinal and psychometric profiles of two cognitive status tests in very old adults. J Clin Psychol.

[9] Xu X (2015). Beyond Screening: Can the Mini-Mental State Examination be Used as an Exclusion Tool in a Memory Clinic?. Diagnostics (Basel).

[10] Schneider LS, Sano M (2009). Current Alzheimer’s disease clinical trials: methods and placebo outcomes. Alzheimers Dement.

[11] Woodford HJ, George J (2007). Cognitive assessment in the elderly: a review of clinical methods. QJM.

[12] Kueper JK, Speechley M, Montero-Odasso M (2018). The Alzheimer’s Disease Assessment Scale-Cognitive Subscale (ADAS-Cog): Modifications and Responsiveness in Pre-Dementia Populations. A Narrative Review. J Alzheimers Dis.

[13] Fujioka T (2016). Gazefinder as a clinical supplementary tool for discriminating between autism spectrum disorder and typical development in male adolescents and adults. Mol Autism.

[14] Kelly M (2017). Technical Report of the Use of a Novel Eye Tracking System to Measure Impairment Associated with Mild Traumatic Brain Injury. Cureus.

[15] Nishizato M, Fujisawa TX, Kosaka H, Tomoda A (2017). Developmental changes in social attention and oxytocin levels in infants and children. Scientific reports.

[16] Fujisawa TX, Tanaka S, Saito DN, Kosaka H, Tomoda A (2014). Visual attention for social information and salivary oxytocin levels in preschool children with autism spectrum disorders: an eye-tracking study. Front Neurosci.

[17] Yamasue, H. *et al*. Effect of intranasal oxytocin on the core social symptoms of autism spectrum disorder: a randomized clinical trial. *Mol Psychiatry* (2018). 10.1038/s41380-018-0097-2 29955161

[18] Boxer AL (2012). Saccade abnormalities in autopsy-confirmed frontotemporal lobar degeneration and Alzheimer disease. Archives of neurology.

[19] Fletcher WA, Sharpe JA (1986). Saccadic eye movement dysfunction in Alzheimer’s disease. Annals of neurology.

[20] Yang Q (2011). Long latency and high variability in accuracy-speed of prosaccades in Alzheimer’s disease at mild to moderate stage. Dementia and geriatric cognitive disorders extra.

[21] Garbutt S (2008). Oculomotor function in frontotemporal lobar degeneration, related disorders and Alzheimer’s disease. Brain: a journal of neurology.

[22] Crawford TJ (2005). Inhibitory control of saccadic eye movements and cognitive impairment in Alzheimer’s disease. Biological psychiatry.

[23] Shafiq-Antonacci R, Maruff P, Masters C, Currie J (2003). Spectrum of saccade system function in Alzheimer disease. Archives of neurology.

[24] Pavisic IM (2017). Eyetracking Metrics in Young Onset Alzheimer’s Disease: A Window into Cognitive Visual Functions. Frontiers in neurology.

[25] Crutcher MD (2009). Eye tracking during a visual paired comparison task as a predictor of early dementia. American journal of Alzheimer’s disease and other dementias.

[26] Fernandez G (2014). Lack of contextual-word predictability during reading in patients with mild Alzheimer disease. Neuropsychologia.

[27] Kawagoe T, Matsushita M, Hashimoto M, Ikeda M, Sekiyama K (2017). Face-specific memory deficits and changes in eye scanning patterns among patients with amnestic mild cognitive impairment. Scientific reports.

[28] Willis SL (2006). Long-term effects of cognitive training on everyday functional outcomes in older adults. JAMA.

[29] Mavros Y (2017). Mediation of Cognitive Function Improvements by Strength Gains After Resistance Training in Older Adults with Mild Cognitive Impairment: Outcomes of the Study of Mental and Resistance Training. J Am Geriatr Soc.

[30] Kivipelto M, Mangialasche F, Ngandu T (2018). World Wide Fingers will advance dementia prevention. Lancet Neurol.

[31] Allgaier M, Allgaier C (2014). An update on drug treatment options of Alzheimer’s disease. Front Biosci (Landmark Ed).

[32] Barnett JH, Lewis L, Blackwell AD, Taylor M (2014). Early intervention in Alzheimer’s disease: a health economic study of the effects of diagnostic timing. BMC Neurol.

[33] Graham WV, Bonito-Oliva A, Sakmar TP (2017). Update on Alzheimer’s Disease Therapy and Prevention Strategies. Annu Rev Med.

[34] Winblad B (2004). Mild cognitive impairment–beyond controversies, towards a consensus: report of the International Working Group on Mild Cognitive Impairment. J Intern Med.

[35] Eramudugolla R (2017). Evaluation of a research diagnostic algorithm for DSM-5 neurocognitive disorders in a population-based cohort of older adults. Alzheimers Res Ther.

