# Supplementary material for: Novel Method for Rapid Assessment of Cognitive Impairment Using High-Performance Eye-Tracking Technology
Source: Sci Rep. 2019 Sep 10;9:12932. doi: 10.1038/s41598-019-49275-x (PMC6736938; doi:10.1038/s41598-019-49275-x)
Supplement: Supplementary file 1 — Supplemental information [file 41598_2019_49275_MOESM1_ESM.docx]

**Supplemental Information**

**Novel Method for Rapid Assessment of Cognitive Impairment Using High-Performance Eye-Tracking Technology**

Akane Oyama^1)^, Shuko Takeda^2)^*, Yuki Ito^2)^, Tsuneo Nakajima^1)^, Yoichi Takami^1)^, Yasushi Takeya^1)^, Koichi Yamamoto^1)^, Ken Sugimoto^1)^, Hideo Shimizu^2),3)^, Munehisa Shimamura^4)^, Taiichi Katayama^5)^, Hiromi Rakugi^1)^, Ryuichi Morishita^2)^*

1) Department of Geriatric and General Medicine, Graduate School of Medicine, Osaka University, Suita, Osaka, 565-0871, Japan.

2) Department of Clinical Gene Therapy, Graduate School of Medicine, Osaka University, Suita, Osaka, 565-0871, Japan.

3) Department of Internal Medicine, Osaka Dental University, Hirakata, Osaka 573-1121, Japan

4) Department of Neurology, Department of Health Development and Medicine, Osaka University, Suita, Osaka 565-0871, Japan

5) Department of Child Development, United Graduate School of Child Development, Osaka University, Kanazawa University, Hamamatsu University School of Medicine, Chiba University, and University of Fukui, Suita, Osaka 565-0871, Japan

**Task movies and pictures for the assessment of cognitive function**

Representative images of each task movie and picture are shown below. See also Methods section in the main text. Images are obtained from JVC KENWOOD Corporation (Japan) with permission.


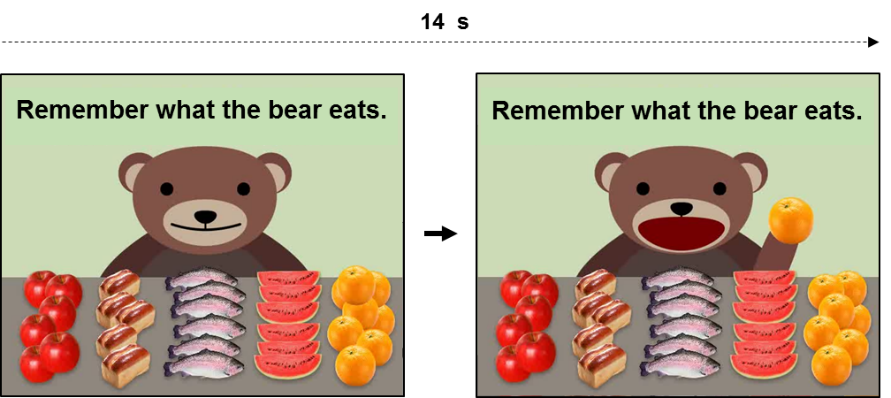


**Task 1-a: Memory task (encoding) (14 s)**


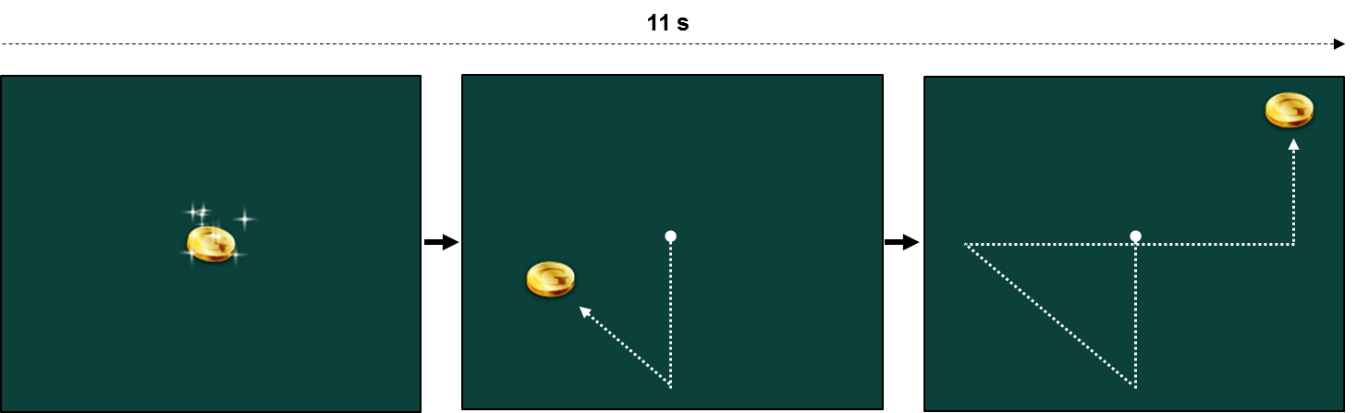


**Task 2: Assessment of smooth pursuit eye tracking (11 s)**

**
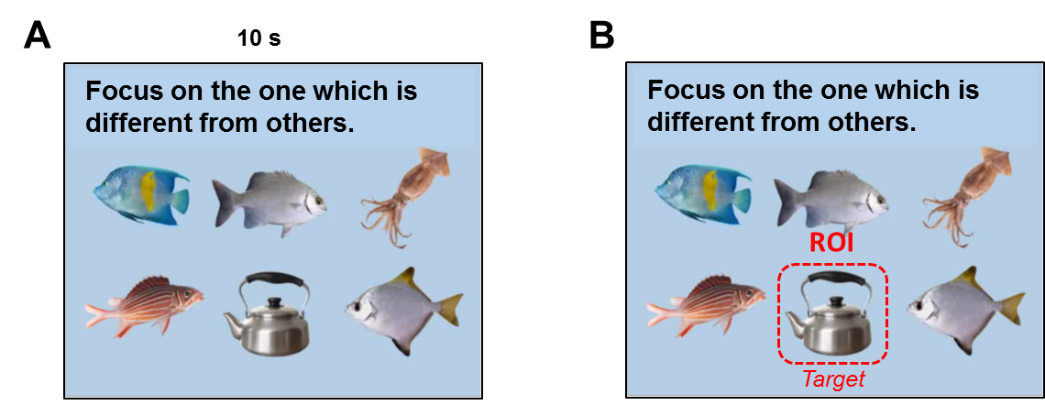
**

**Task 3: Deductive reasoning (odd one task) (10 s)**

(A) The picture displayed on the monitor. (B) ROI on the correct answer (target) is overlaid on the image.


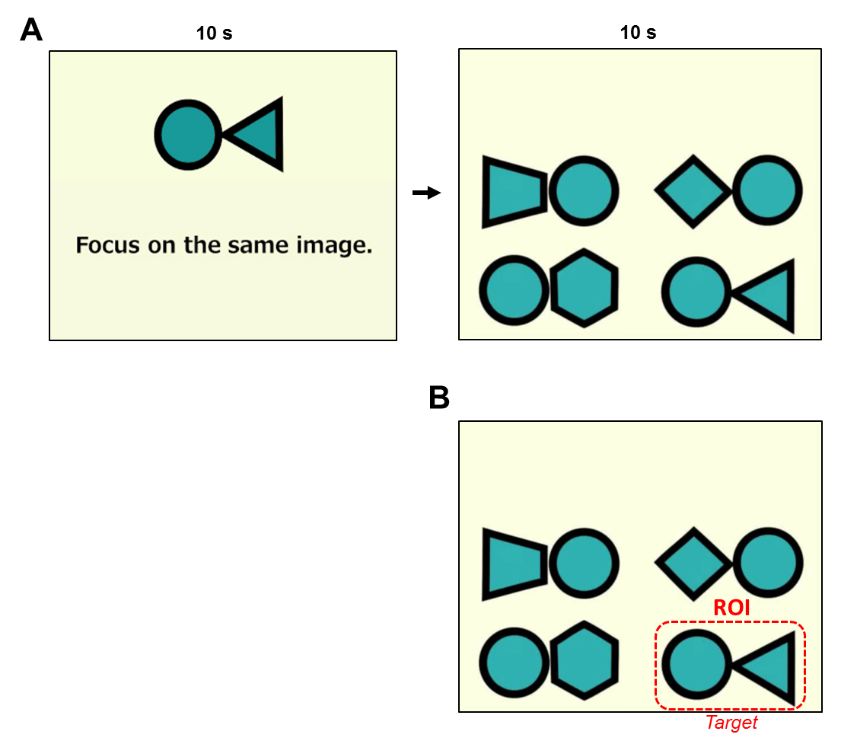


**Task 4: Visual working memory task 1 (pattern matching) (20 s)**

(A) The pictures displayed on the monitor. (B) ROI on the correct answer (target) is overlaid on the image.

**
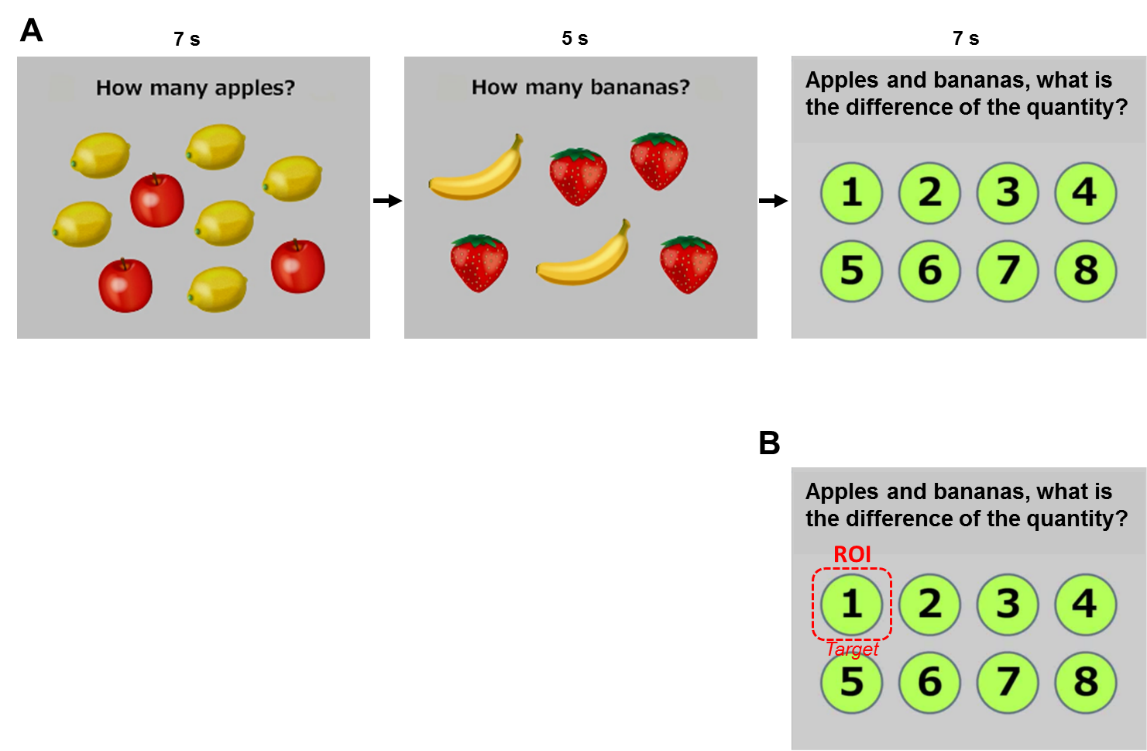
**

**Tasks 5–7: Attention and calculation task (56 s in total)**

(A) The pictures displayed on the monitor. (B) ROI on the correct answer (target) is overlaid on the image.

**
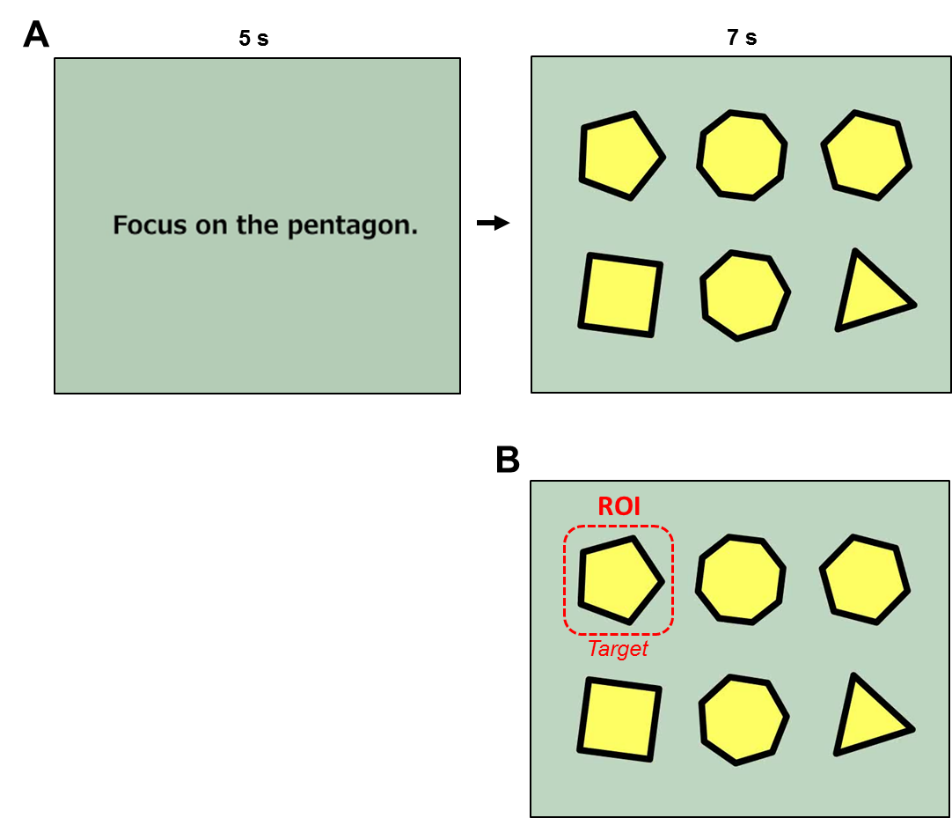
**

**Tasks 8–9: Visuospatial function task (24 s in total)**

(A) The pictures displayed on the monitor. (B) ROI on the correct answer (target) is overlaid on the image.

**
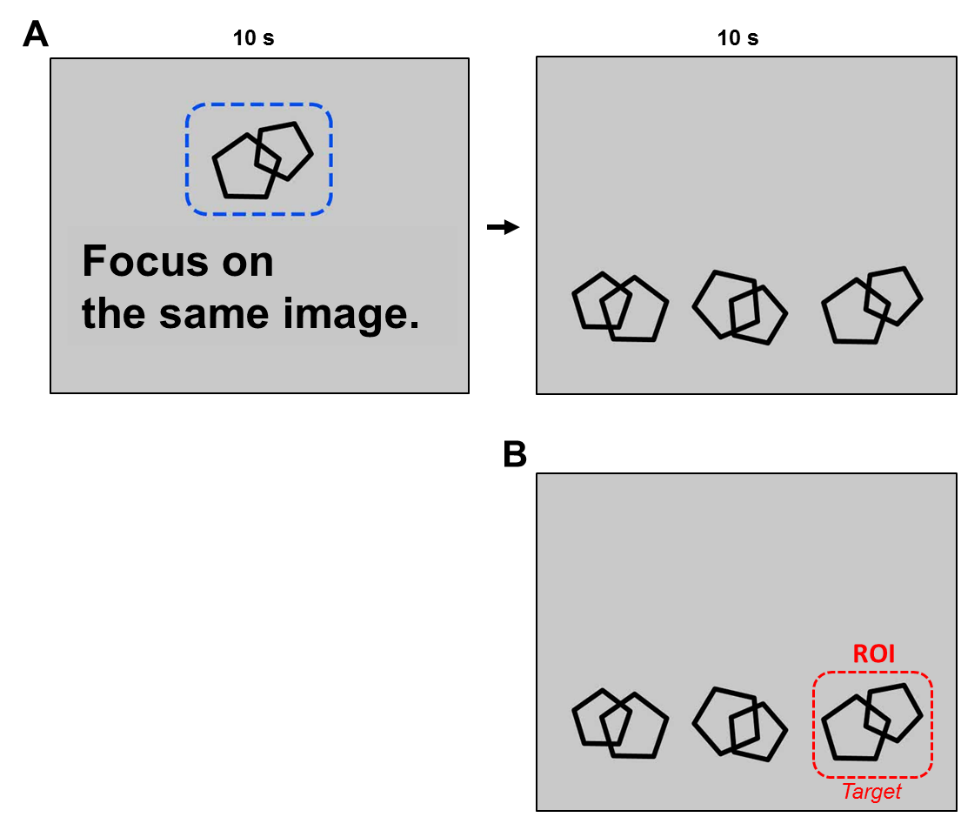
**

**Task 10: Visual working memory task 2 (intersecting double pentagon) (20 s in total)**

(A) The pictures displayed on the monitor. (B) ROI on the correct answer (target) is overlaid on the image.


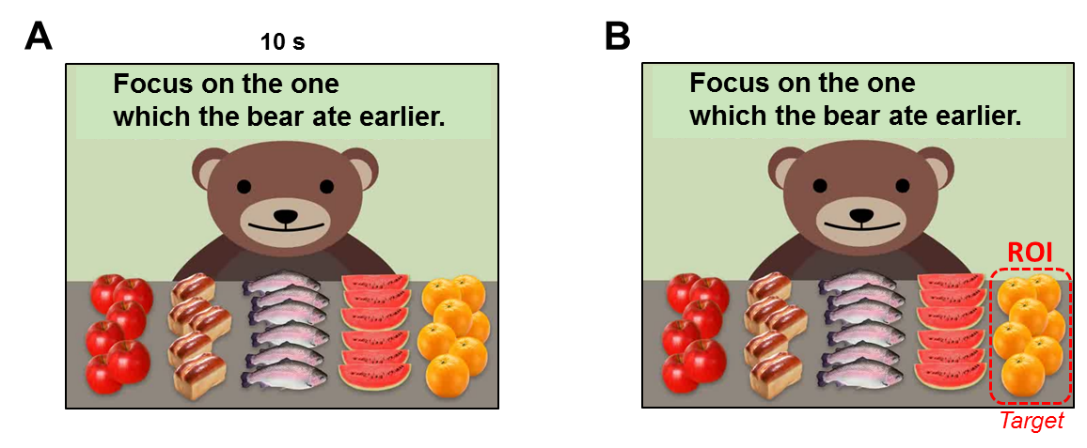


**Task 1-b: Memory task (recall) (10 s)**

(A) The movie displayed on the monitor. (B) ROI on the correct answer (target) is overlaid on the image.

**
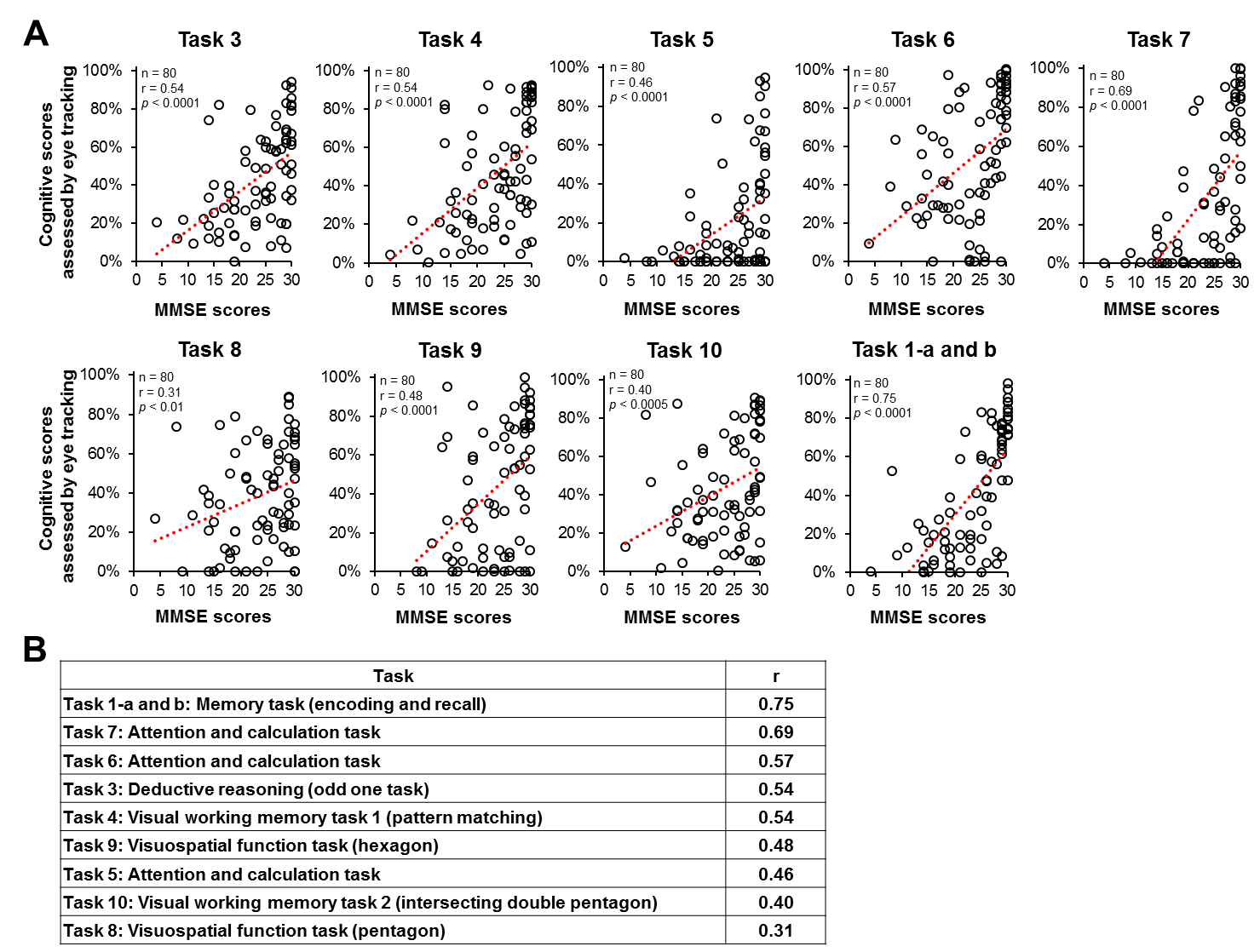
**

**Supplemental figure 1**

Correlation between MMSE scores and eye tracking-based cognitive scores in each task movie or picture. (A) Scattered plots of MMSE scores vs. eye tracking-based cognitive scores in each task. (B) Tasks are ordered in decreasing level of correlation according to Spearman’s coefficient r value.


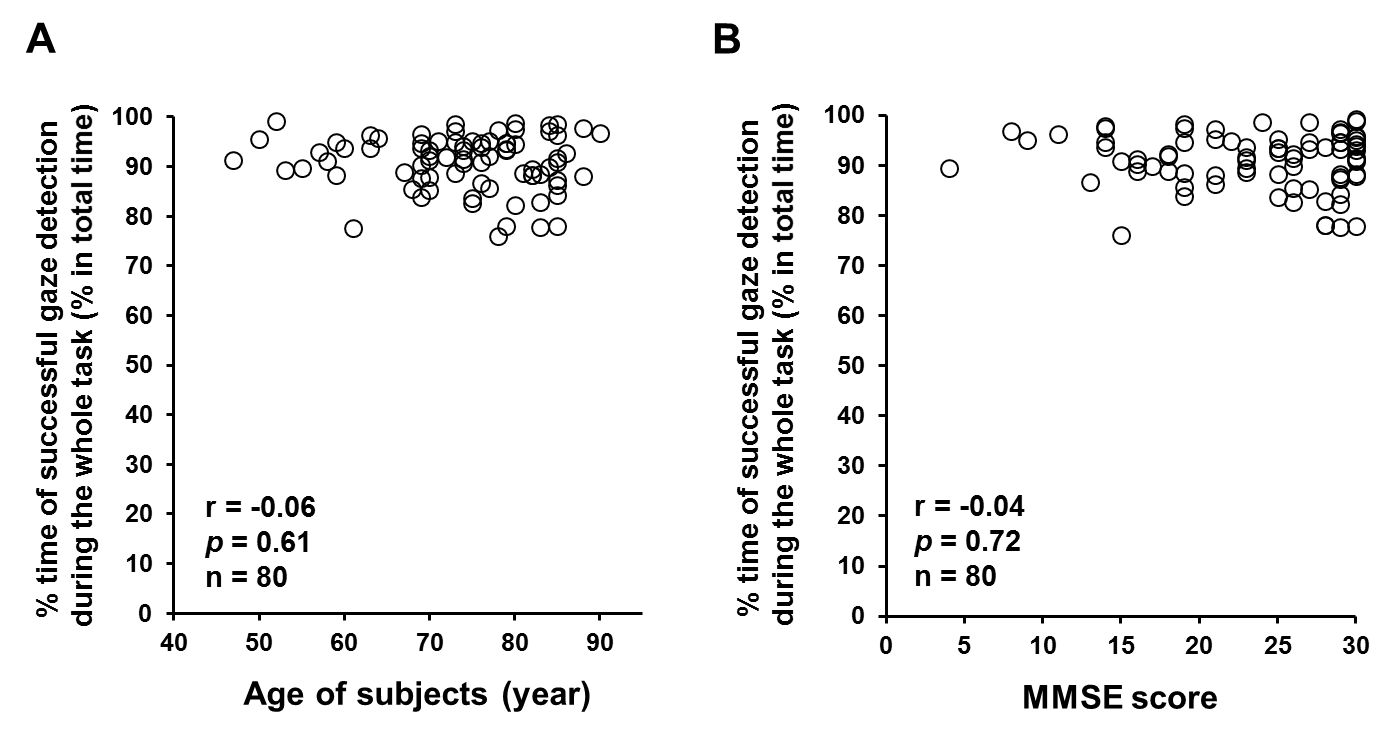


**Supplemental figure 2**

**Successful gaze detection in the elderly and subjects with severe cognitive impairment.** (A) The correlation between the age of subjects and the % time of successful gaze detection during the whole task (= 178 s). Age does not affect the efficiency of gaze detection. *p* = 0.61, Spearman’s rank test, n = 80. (B) The correlation between the MMSE scores of subjects and the % time of successful gaze detection during the whole task. Cognitive impairment does not affect the efficiency of gaze detection. *p* = 0.71, Spearman’s rank test, n = 80. MMSE, Mini–Mental State Examination.


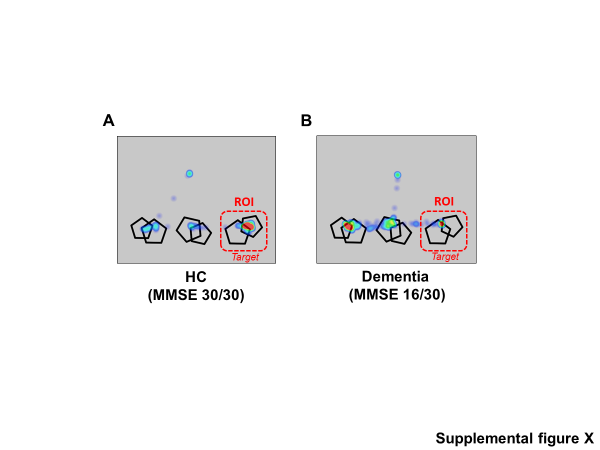


**Supplemental figure 3**

**Duration-based heatmap images of eye movements during cognitive tasks.** Representative heatmap images obtained from HC (A) and dementia (B) during the visual working memory task (Task 10). (A) HC subject (MMSE 30/30) successfully remembered the target image during the encoding session and was able to focus on the same image (right) during the recall session. (B) Subject with dementia (MMSE 16/30) showed a low frequency of fixation to the target image and a higher frequency to the non-target images (left and middle). HC, healthy control; MMSE, Mini–Mental State Examination; ROI, region of interest.
